# Microbial stars: shedding light on gut microbes’ role in insulin resistance and innovative diabetes therapies

**DOI:** 10.1080/19490976.2024.2307581

**Published:** 2024-01-26

**Authors:** Jinwei Zhang

**Affiliations:** a Institute of Biomedical and Clinical Sciences, Medical School, Faculty of Health and Life Sciences, University of Exeter, Exeter, UK; b State Key Laboratory of Chemical Biology, Research Center of Chemical Kinomics, Shanghai Institute of Organic Chemistry, Chinese Academy of Sciences, Shanghai, China

**Keywords:** Gut microbes, insulin resistance, metabolic syndrome, type 2 diabetes mellitus, therapies

## Abstract

The role of gut microbiota in insulin resistance (IR), Metabolic Syndrome (MetS), and Type 2 Diabetes Mellitus (T2DM) is rapidly gaining recognition. However, the mechanisms and implications of gut bacteria in these conditions remain enigmatic. This commentary not only highlights the findings of a recent multi-omics study by Takeuchi et al. but also offers a unique perspective by integrating personal opinions and insights. The discussion revolves around the intricate connection between gut microbes and IR, suggesting novel therapeutic potential in targeting gut microbial carbohydrate metabolism for improved IR management and metabolic health.

## The gut microbiome and insulin resistance

Insulin, a key hormone, regulates blood glucose levels by promoting glucose uptake in muscles and the liver.^1^ Within our gastrointestinal tract, trillions of bacteria break down carbohydrates, a process long suspected to be linked to IR and metabolic disorders like obesity and pre-diabetes.^2,3^ However, the mechanisms remained unclear due to the gut’s bacterial diversity and limited metabolic data. In a recent Nature publication, Takeuchi et al.^4^ This study also connected fecal metabolites to Metabolic Syndrome (MetS), closely related to IR. This commentary discusses the emerging role of gut microbes in insulin resistance (IR), highlighting their intricate connections to metabolic disorders, and providing a critical appraisal of the study’s strengths and limitations.

## A multi-omics approach to unravel the gut microbiota-IR connection

**Figure 1.**
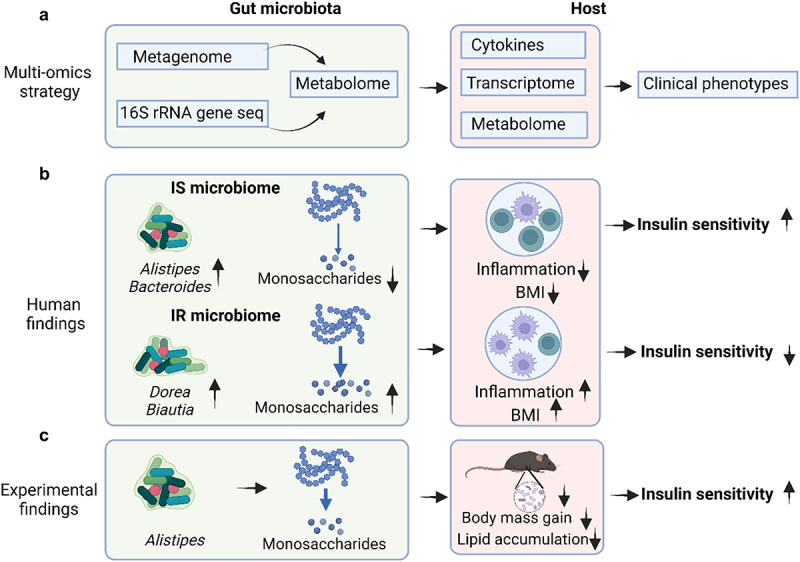
Gut microbiota carbohydrate metabolism’s role in insulin resistance. A. This study combines fecal metabolome, 16S rRNA gene sequencing, and metagenome data with host metabolome, transcriptome, and cytokine data to explore the role of gut microbiota in insulin resistance (IR). B. monosaccharides and carbohydrate degradation products are significantly elevated in IR. Metagenomic findings reveal enhanced poly- and disaccharide breakdown and utilization in IR, closely linked to fecal monosaccharides. These metabolites may influence host metabolic parameters, such as BMI, partially through specific cytokines. C. animal experiments show that oral administration of *alistipes indistinctus*, selected based on human cohort findings, reduces intestinal carbohydrate and lipid accumulation, ameliorating IR. Diagram created using BioRender.com, with figure elements adapted from Turnbaugh et al.^4^ *abbreviations: IR, insulin resistance; 16S rRNA, 16S ribosomal ribonucleic acid; BMI, body mass index.*

Takeuchi et al. conducted a comprehensive study that integrated unbiased fecal metabolomic profiles, metagenomic data, and host transcriptomic information (Figure 1A). The study cohort comprised 306 individuals aged 20 to 75, all of whom participated between 2014 and 2016 in regular annual health checkups at the University of Tokyo Hospital. Among the participants, 112 had normal metabolic profiles, 100 were obese, and 101 were prediabetic. Various assessments, including physical exams, laboratory tests, fecal sample collection for 16S ribosomal ribonucleic acid (rRNA) pyrosequencing and metabolomic analysis, and blood sampling for serological metabolomic analyses, were conducted. Exclusion criteria included confirmed diabetes diagnoses, habitual use of diabetes or intestinal disorder medications, recent antibiotic use within two weeks of sample collection, and a weight loss exceeding three kilograms in the preceding three months. IR was assessed using the homeostatic model assessment of IR (HOMA-IR) with a threshold score of 2.5 or higher.

To analyze gut microbial activities, fecal metabolites and predicted genes were categorized into co-abundance groupings (CAGs) and Kyoto Encyclopedia of Genes and Genomes (KEGG) categories. Transcriptomic data from peripheral blood mononuclear cells (PBMCs) were obtained using the cap analysis of gene expression (CAGE) approach to quantify gene expression at the transcription-initiation-site level. Receiver operative curve (ROC) analyses with area under the curve (AUC) values were conducted using the random-forest classifier system to evaluate the potential of omics data from fecal samples in predicting IR. Fecal 16S rRNA gene sequencing, metagenome sequencing, metabolomic data, and their combined datasets were used to identify estimator variables for modeling via the maximum-relevance minimum-redundancy algorithm. Correlation and regression studies involving clinical indicators adjusted for significant confounding variables, such as gender and age. Hydrophilic metabolites in fecal and serological samples were quantified using gas chromatography-tandem mass spectrometry (GC-MS/MS) techniques.

## Key study findings

The study revealed that the majority of IR-correlated hydrophilic metabolites within CAGs were associated with carbohydrate metabolism, mainly monosaccharides (Figure 1B). IR also linked to elevated short-chain fatty acid (SCFA) levels, particularly propionate, in line with its function in gluconeogenesis.^5^ This also aligns with prior research indicating that the dietary provision of SCFAs shielded mice from obesity and IR induced by diet.^6–8^ Certain bacterial species in the gut are known to ferment dietary fiber, resulting in the production of metabolites such as SCFAs.^9^ A KEGG pathway enrichment analysis of these metabolites emphasized their association with carbohydrate metabolism. Notably, galactose, fructose, xylose, and mannose significantly correlated with IR. An abundance of monosaccharides has the capacity to encourage the accumulation of lipid in abnormal locations, along with triggering immune cells, resulting in mild inflammation, resulting in low-grade inflammation and IR.^10–12^ Specifically, fructose is widely recognized as a key contributor to inflammation and IR due to its involvement in lipid accumulation,^13^ whereas galactose has been observed to play a role in the energy metabolism of activated immune cells.^14^ Additional studies have shown that a fructose-enriched liquid diet induces inflammation and IR in the visceral adipose tissue of adult female rats,^15^ and that IR resulting from fructose infusion may contribute to the increased production of triglyceride-rich lipoproteins in the intestine.^16^ Furthermore, prolonged exposure to fructose, despite not immediately raising insulin levels, is suggested to indirectly lead to hyperinsulinemia and obesity through alternative mechanisms, including the fructose transporter GLUT5.^17^ Bacteroidal species efficiently absorbed carbohydrates, fueling fermentation product synthesis. In mice, *Alistipes indistinctus* presence reduced IR and altered intestinal glucose metabolites, consistent with human findings.

The study also showed that higher IR related to a predominance of “c”-order bacteria in gut microbiomes and elevated fecal carbohydrate levels, primarily due to *Lachnospiraceae*. Conversely, participants with more *Bacteroidales-*type bacteria had lower IR and reduced fecal monosaccharides. To assess these bacteria’s direct impact, experiments in culture and mice were conducted. *Bacteroidales* bacteria consumed monosaccharides like those in high-IR feces. *A. indistinctus* exhibited the broadest monosaccharide consumption range. In obese mice, *A. indistinctus* lowered blood sugar levels, mitigated IR, and reduced carbohydrate availability.

### Alistipes indistinctus

*Alistipes* is a recently discovered bacterial genus, primarily found in medical clinical samples, albeit at a lower frequency compared to other Bacteroidetes genera commonly associated with chronic intestinal inflammation.^18^ *A. indistinctus*, identified in 2010, is a Gram-negative bacterium commonly found in the human intestinal microbiota and isolated from feces.^19^ It exhibits some differences from other *Alistipes* species, such as *A. putredinis*^20^ or *A. onderdonkii*.^21^ Colonies of *A. indistinctus* on modified GAM agar are typically circular, measuring 0.1–0.5 mm in diameter, with a slightly opaque and gray appearance. Its primary metabolic products in PYG broth are succinic and acetic acid, with iso-C15:0 as the dominant cellular fatty acid.^19^ Its complete genome sequence has been reported recently.^22^ A recent study involving a dietary intervention with high-amylose maize (HAM) reported several beneficial outcomes, including reduced body weight gain, adipocyte hypertrophy, and dyslipidemia.^23^ This intervention also alleviated nonalcoholic fatty liver disease, IR, impaired glucose tolerance, and inflammation in obese mice fed a high-fat diet. At the species level, *A. indistinctus* significantly increased in response to this HAM dietary intervention.^23^

## A critical appraisal of the study

The study by Takeuchi et al. is a remarkable example of how multi-omics approaches can shed light on the complex interactions between gut microbiota and host metabolism. The study provides novel insights into the role of gut microbial carbohydrate metabolism in IR, a fundamental mechanism linked to MetS and T2DM. The study also demonstrates the potential of fecal metabolomics as a diagnostic tool for IR and a source of biomarkers for metabolic health. Moreover, the study identifies *A. indistinctus* as a promising candidate for modulating gut microbiota and improving IR in obese mice, suggesting a therapeutic avenue for human metabolic disorders.

However, the study also has some limitations that warrant further investigation. First, the study cohort was relatively small and homogeneous, consisting of Japanese individuals from a single hospital. Therefore, the generalizability of the findings to other populations and settings may be limited. Second, the study did not account for other factors that may influence gut microbiota and IR, such as diet, lifestyle, genetics, and environmental exposures. Third, the study did not establish a causal relationship between gut microbiota and IR, but rather a correlation based on cross-sectional data. Longitudinal and interventional studies are needed to confirm the direction and magnitude of the effect of gut microbiota on IR. Fourth, the study did not explore the molecular mechanisms underlying the observed associations between gut microbial carbohydrate metabolism and IR. For instance, how do fecal monosaccharides and SCFAs affect host glucose homeostasis, inflammation, and insulin signaling? How do gut microbiota interact with host immune cells and cytokines? How do *A. indistinctus* and other *Bacteroidales* bacteria confer protection against IR? These questions remain to be answered by future studies.

## Conclusions and perspective

Gut microbes have emerged as promising targets for addressing IR and associated metabolic disorders, including obesity and T2DM. Takeuchi et al.‘s study provides fresh perspectives on the intricate link between gut microbial carbohydrate metabolism and IR, proposing that targeting this metabolism could serve as a viable therapeutic strategy for improving metabolic health. However, the field presents both challenges and opportunities for future research and innovation.

To advance our knowledge, it is crucial to delve deeper into the molecular mechanisms underlying the gut microbiota’s impact on host metabolism and inflammation, considering variations across diverse populations and settings. Precision in developing interventions that modulate gut microbial carbohydrate metabolism and enhance insulin sensitivity without adverse effects is paramount. Long-term safety and efficacy assessments, comparing cost-effectiveness and feasibility with standard treatments, are essential.

Achieving these goals requires integration of multi-omics approaches, such as metagenomics, metabolomics, transcriptomics, and proteomics, to capture the complexity of the gut microbiome and its interactions. Advanced computational tools are necessary for analyzing the vast and heterogeneous data generated. Collaborations among researchers, clinicians, patients, and policymakers are essential for translating basic research findings into clinical practice and public health policy.

In conclusion, Takeuchi et al.‘s study marks a significant advancement in comprehending the role of gut microbes in IR and metabolic disorders. Yet, there is substantial work ahead to fully harness the therapeutic potential of gut microbes. Embracing the challenges and opportunities in this field can pave the way for precise, personalized, and effective interventions, ultimately improving the lives of millions affected by metabolic disorders globally.
